# Evaluation of larval surface antigens from infective larvae of *Strongyloides venezuelensis* for the serodiagnosis of human strongyloidiasis

**DOI:** 10.1590/S1678-9946202466001err

**Published:** 2024-01-05

Rev Inst Med Trop Sao Paulo. 2023;65:e1

http://doi.org/10.1590/S1678-9946202365001

On page 1, Abstract:

Where it reads:

At a second cut-off value (OD 450 nm = 0.286), sensitivity and specificity were 70% and 100%, respectively, with a diagnostic accuracy of 0.91. However, at an alternative third cut-off value (OD 450 nm = 0.589), sensitivity and specificity were 95% and 97.8%, respectively, with a diagnostic accuracy of 0.97.

Should be read:

At a second cut-off value (OD 450 nm = 0.589), sensitivity and specificity were 70% and 100%, respectively, with a diagnostic accuracy of 0.91. However, at an alternative third cut-off value (OD 450 nm = 0.286), sensitivity and specificity were 95% and 97.8%, respectively, with a diagnostic accuracy of 0.97.

